# Comparison of a digital iodine-specific dietary screener with 24-hour recall and urinary iodine concentration

**DOI:** 10.1017/jns.2023.74

**Published:** 2023-08-01

**Authors:** Hanne Rosendahl-Riise, Siri Aksnes, Zoya Sabir, Ellen K. Ulleberg, Thea Myklebust-Hansen, Inger Aakre

**Affiliations:** 1 Mohn Nutrition Research Laboratory and Center for Nutrition, Department of Clinical Medicine, University of Bergen, Bergen, Norway; 2 Department of Clinical Medicine, University of Bergen, Bergen, Norway; 3 Norwegian Dairy Council, Oslo, Norway; 4 Institute of Marin Research, Bergen, Norway

**Keywords:** 24-hour recall, Dietary screener, Iodine, Urinary iodine concentration, Validation

## Abstract

Mild-to-moderate iodine deficiency remains a problem worldwide, including in Norway. Of particular, concern is fertile, pregnant and lactating women. The Norwegian Dairy Council developed a digital iodine-specific dietary screener (I-screener) for the assessment of iodine intake levels but has yet to be validated. The aim was thus to investigate the relative validity of the I-screener by comparing estimates of iodine intake from the I-screener against a single 24-hour recall (24HR) and urinary iodine concentration (UIC) in fertile women. Healthy females were recruited in Bergen in August–December 2021. Six spot-urine samples from six consecutive days were collected into a pooled sample to assess UIC. Each participant completed a single administration of the I-screener and the 24HR. The estimated daily iodine intake from the I-screener was compared with the estimations from the 24HR and UIC. Seventy-two women aged 19–39 completed the study. The median UIC was 76 μg/l. Compared with the 24HR, the I-screener placed 83 % of the participants in the same/adjacent tertial, with a slight agreement between the methods (Cohen's kappa = 0⋅187). The present study shows an acceptable correlation between the I-screener and the 24HR (*r* = 0⋅318), but not between the I-screener and UIC (*r* = 0⋅122). Despite its varying iodine estimate abilities, the I-screener may be used as an initial screening tool to rank fertile women on an individual level into deficient inadequate, and sufficient iodine intake. However, due to the relatively high risk of misclassification, further assessment of iodine status should follow.

## Introduction

Through the iodine fortification of salt applied in many countries, severe iodine deficiency has almost been eliminated^(1)^. However, mild-to-moderate iodine deficiency remains a problem in many parts of the world, including Norway^(2)^. Mild-to-moderate iodine deficiency has been identified in several population groups including women of fertile age^(3,4)^ and pregnant women^(5,6)^ which is of concern as the fetus and infant are dependent on adequate iodine supply for optimal development^(7)^.

In Norway, the most important sources of iodine are milk, dairy products, eggs and marine white fish. The content of iodine is naturally low in milk, dairy products and eggs, but due to the iodine fortification of animal fodder since the 1950s, these foods are good sources of iodine^(8)^. While iodised salt is an important iodine source in many countries, the table salt in Norway is fortified with insignificant amounts of iodine (5 μg iodine/g salt)^(9)^. A measure that has been introduced for Norwegian women of fertile age is the recommendation of an iodine supplement before pregnancy if the dietary intake of milk, dairy products and marine white fish is low^(10)^.

Median UIC is considered the most efficient method for assessing iodine status in a population. As iodine deficiency or excess may be one of the most important environmental factors that may cause thyroid dysfunction, thyroid hormones TSH, fT3 and fT4 can be used as indicators of thyroid function, however, these are not considered sensitive markers of iodine nutrition^(11)^. Increased Tg levels can result from increased thyroid cell mass and TSH stimulation which can be seen in an iodine-deficient population, however, whether it can be used as an individual indicator for iodine status is still uncertain as there is a lack of established reference values^(11)^. Measurement of the thyroid size may be used for assessing the severity of iodine deficiency in a population, but with mild or moderate iodine deficiency, goitre is often not present or does not reflect current status^(12)^.

As there are several challenges concerning the existing biomarkers for assessing iodine status, such as intra- and inter-variability of UIC and low sensitivity of TSH, fT3 and fT4^(12)^, dietary assessments of iodine intake may be a useful tool to identify common dietary sources of iodine and estimate the intake of iodine. The dietary sources of iodine are limited, thus quantification and recall of a person's dietary sources are relatively easy. That means that dietary assessment is a suitable method for assessing iodine status at an individual level. The validity of a dietary assessment method refers to whether the method measures what it is intended to measure^(13)^ or assesses the agreement with a gold standard method. However, there is no gold standard method available for assessing the validity of a dietary screener or an FFQ, hence it is recommended to use more than one reference method to add credence to the results^(14)^.

Considering the current situation of mild-to-moderate iodine deficiency in vulnerable groups, it is essential to have feasible and cost-effective tools that may be used in the assessment of iodine status. The Norwegian Dairy Council (Melk.no)^(15)^ developed a publicly accessible digital iodine-specific dietary screener (I-screener) for the assessment of the iodine intake level (deficient, insufficient or adequate intake) on an individual level. By examining iodine intake easily and cost-effectively, the I-screener has the prerequisite to contribute to the assessment of iodine nutrition in the Norwegian population, and could be an opportunity for other countries to follow. Thus, the aim of the present study is to compare estimated iodine intake by the I-screener with estimated iodine intake from a single 24-hour recall (24-HR) and UIC from six spot-urine samples in women of reproductive age.

## Method

### Study design and subjects

The present study is based on primary data collection, and the recruitment of participants was performed in August–December 2021 in Bergen, Norway. The recruitment of participants was completed through social media platforms, posters on study campuses, gyms, local stores and at the University of Bergen (UiB) and Western Norway University of Applied Sciences. Some participants were recruited through snowball sampling^(16)^.

The inclusion criteria for the study were healthy women aged 18–40 years. In addition, the participants had to possess sufficient language skills to be able to complete the questionnaires in Norwegian and be able to meet physically at the study centre in Bergen. The exclusion criteria included being pregnant, lactating or having known thyroid disease. The participants were enrolled in the study by filling out an online form with their contact information and confirming their eligibility to participate based on the inclusion and exclusion criteria. They were further contacted by email to schedule the study visits.

The study was conducted at the Research Unit for Health Surveys (RUHS), a core facility at the UiB and Haukeland University Hospital. The trained personnel, consisting of bioengineers and nurses, assisted with the laboratory training of the investigators and took blood samples from the participants.

### Data collection methods

**Fig. 1.**
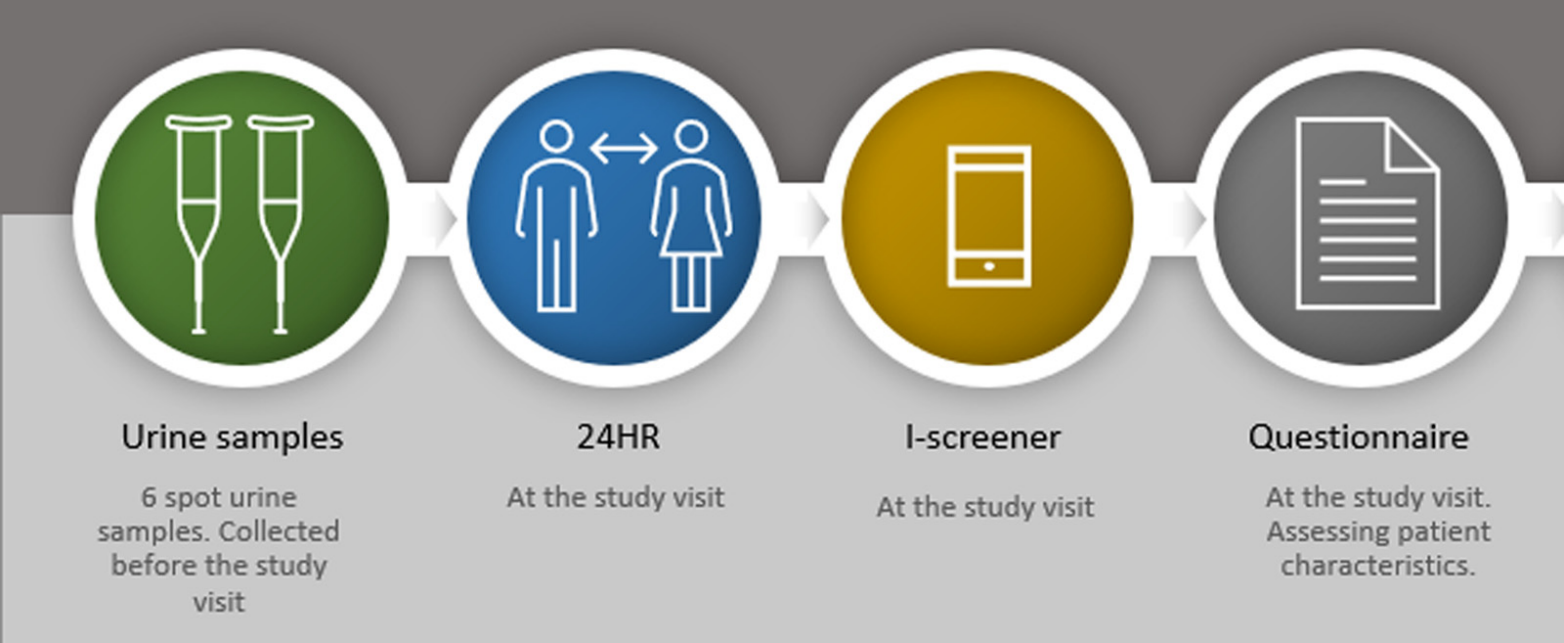
An overview of the timeline of the study.

The data collected in this study consisted of six spot-urine samples collected from six consecutive days, which were completed at home before the study visit. During the study visits, the participants provided a blood sample and completed one administration of the digital I-screener and one 24-hour recall (24HR) interview. A questionnaire was handed out to the participants to obtain socio-demographic variables such as age, body weight, height, education and nicotine use. An overview of the timeline of the study is provided in Fig. 1.

#### Digital iodine-specific dietary screener (I-screener)

The I-screener is an online tool publicly available and free of charge that is developed by the Norwegian Dairy Council^(15)^. The I-screener consists of sixteen items and inquiries about the intake of thirteen iodine sources (see Supplementary Table S1 for an overview of the items). The Norwegian Food Composition Table is used in iodine intake calculations^(17)^. Introductory questions in the I-screener inquire about gender, age, pregnancy and lactation, to allow the I-screener to adjust the result according to the iodine requirements of the subject. The I-screener aims to assess whether the iodine intake of the subject is sufficient or insufficient according to the Nordic Nutrition Recommendations^(18)^, based on the habitual weekly intake. The results are grouped into three categories, and the results from the I-screener are given by a feedback message based on which category the subject is placed into (Table 1). The exact daily estimated iodine intake values are not provided on the result page but can be found in the web address/URL^(19)^. The participants were asked to complete the digital I-screener once during the study visit. The result of the I-screener in μg/d was reported as an estimated daily iodine intake for comparison with the reference methods.

**Table 1.** An overview of the three categories of iodine intake for adults (not pregnant, nor breast-feeding) generated by the digital iodine-specific food frequency questionnaire (I-screener)

Iodine intake category	The messages generated in the I-screener
<100 μg/d	‘You are probably not consuming enough iodine, and should increase your dietary intake’
100–150 μg/d	‘You are probably consuming an inadequate amount of iodine and should increase your dietary intake’
>150 μg/d	‘It looks like you are consuming adequate amounts of iodine’

#### 24-hour recall

The dietary reference method in this study was a single administration of the 24HR. The 24HRs were conducted by an in-person interviewer using a standard multiple-pass method by two trained study personnel. The iodine intake from the 24HR was estimated using the dietary calculation tool ‘Kostholdsplanleggeren’^(20)^, which uses food composition data from the Norwegian Food Composition Table^(17)^.

#### Urinary iodine concentration

The equipment (Sarstedt urine glasses) for the six spot-urine samples from six consecutive days was handed out to the participants before the study visit. Participants were instructed to collect spot-urine samples at any point except for the first voiding of the day. The samples were stored in a refrigerator or freezer to avoid excessive bacterial growth until delivery to the study centre. On the day of the study visit, the participants brought the urine samples with them. In the lab, 4 ml of each spot-urine sample was homogenised before being pipetted into a tube for a pooled sample (Sarstedt 50 ml screw cap tube) with the use of an automatic pipette (Sarstedt), and further homogenised, before 10 ml was pipetted into a cryotube (Sarstedt 15 ml screw cap tube) and stored at −20 °C pending analysis.

At the end of the study, the urine samples were analysed at the Institute of Marine Research (IMR) by inductively coupled plasma mass spectrometry (ICP-MS). For the determination of iodine, 500 μl urine was diluted in 4⋅5 ml 1 % tetramethylammonium hydroxide (TMAH) and filtered using a sterile membrane with a 0⋅45 μm pore size and a single-use syringe. The samples were analysed to a urine calibration curve (standard addition curve). Internal validity of the method was verified with certified reference material (SRM); 22 Seronorm Trace Elements Urine. The measurement uncertainty for iodine is 20 % for the whole measurement range.

#### Estimating daily iodine intake from urinary iodine concentration

Estimated daily iodine intake from UIC (E-UIC) was calculated using the following equation: UIC (μg/l) × 0⋅0235 × body weight (kg)^(21)^. Self-reported body weight was used for the estimation. The WHO epidemiological criteria were applied for the evaluation of the median UIC values^(12)^.

### Ethics

The study was approved by the Regional Committees for Medical and Health Research Ethics West (REK-vest 2021/232247). The study was conducted and performed according to the Declaration of Helsinki. Participation was voluntary and signed written consent was provided by all participants. The participants could withdraw from the study at any time without further explanation. The study is registered in the public trial registry Clinicaltrials.gov (2021/232247).

### Statistical analysis and presentation of data

Descriptive statistics are reported as frequency (%) for categorical variables. Variables were tested for normality by using the Kolmogorov–Smirnov test, and by visual inspection of Q-Q plots and histograms. For continuous variables, mean (sd), median (IQR) and min-max are reported. The difference between estimated iodine intake from the I-screener, 24HR and UIC was assessed using Wilcoxon signed-rank test. The relative validity of the I-screener was assessed by using Spearman's rank order correlations coefficient (Spearman's rho), reflecting the degree of which the I-screener, the 24HR, UIC and estimated iodine intake from UIC ranked participants equally in terms of estimated iodine intake. In accordance with other comparison studies^(22)^, the criteria from Landis and Koch^(23)^ were used to assess agreement, where a *k* of 0⋅01–0⋅20 represents a slight agreement, 0⋅21–0⋅40 fair agreement, 0⋅41–0⋅60 moderate agreement, 0⋅61–0⋅80 substantial agreement and 0⋅81–1⋅00 almost perfect agreement. Cross-classification tables were created to evaluate the extent to which the I-screener classified participants into the same tertile of iodine intake as the 24HR, UIC and iodine intake from UIC. The stability or agreement between methods is presented as numbers and percentage of participants remaining in their tertile (stable tertile, stable/adjacent tertile or opposite tertile) (one tertile between, e.g., from first to the third tertile), compared to the 24HR, UIC and iodine intake from UIC.

Bland–Altman plots were used to analyse the agreement between methods, using a plot of the mean difference of iodine intake between the two methods against the mean iodine intake of the two methods, also showing 95 % limits of agreement (LOA). This was applied to graphically assess the presence of bias or disagreement^(24)^. Unadjusted and adjusted (BMI, age, education level and nicotine use) coefficients are presented. Data analysis was performed using Statistical Package for Social Sciences version 2, IMB Corporation (IBM Corp. Released 2020. IBM SPSS Statistics for Macintosh, Version 27.0. Armonk, NY: IBM Corp).

## Results

### Study population

**Fig. 2.**
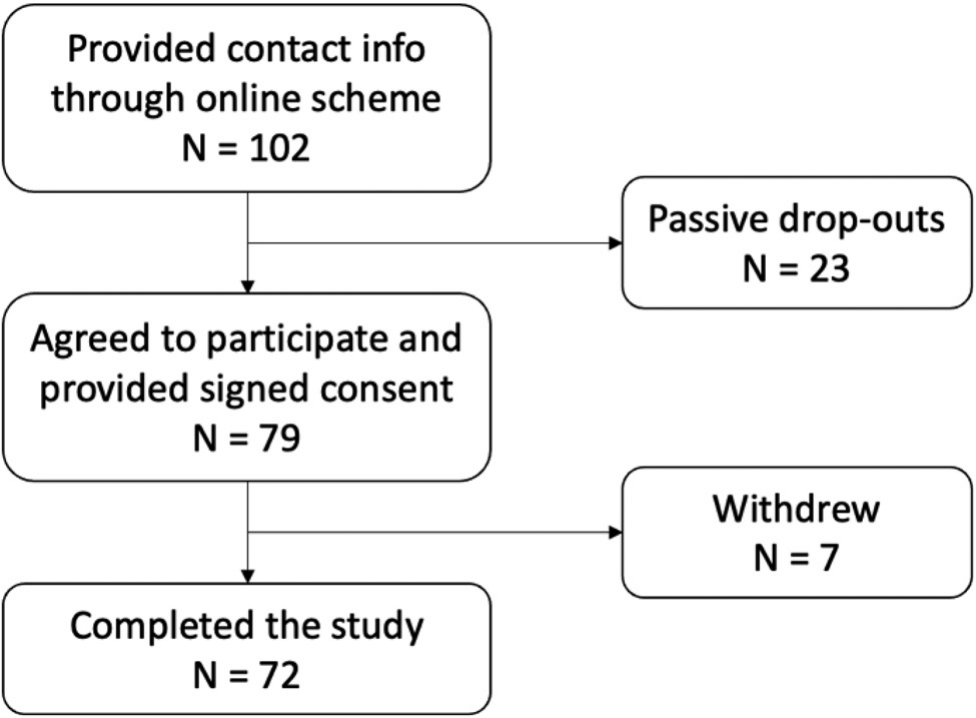
Overview of the participation flow in the project.

Of the 102 interested, a total of 79 women agreed to participate. During the study, seven participants chose to withdraw due to the following reasons: illness during the spot-urine collecting period on the study visit day or personal reasons. An overview of the participation flow is found in Fig. 2.

### Participant characteristics

The participant characteristics of the women enrolled in the study are shown in Table 2. The study included seventy-two women between the ages of 19–39 years. Out of the seventy-two women, forty were students, and twenty-six were employed in various occupations. More than 50 % of the population studied or worked in health-related fields. None of the participants smoked, but five used non-smoke tobacco [snus] daily and one on occasion.

**Table 2.** Baseline characteristics of participants (*n* 72)

Variable, unit	Mean (sd)	Min, Max
Age (years)	27 (6)	19–39
Weight (kg)	65 (9)	45–90
Height (cm)	168 (6)	156–182
BMI (kg/m^2^)	23 (3)	17–32

Variable	*N*	Percent
Marital status		
In a relationship	41	57
Single	31	43
Country of birth		
Norway	64	89
Other	8	11
Education level, *n*		
High school/diploma degree	23	32
1–4 years of college/university	22	31
>4 years of college/university	27	37
Non-smoke tobacco (snus)		
Sometimes	1	1
Daily	5	7
Planning pregnancy within 2 years		
Don't know	21	29
Yes	5	7

#### Iodine status

The median UIC in the study population was 76 μg/l (see Table 3). 11 (15 %) of the participants had UIC values below 50 μg/l (data not shown).

**Table 3.** Baseline urinary iodine concentration (UIC) from spot-urine samples and estimated iodine intake from the iodine-specific food frequency questionnaire 24HR (μg/d), and estimated iodine intake from urinary iodine concentration (E-UIC, μg/d) (*n* 72)

Biomarker, unit	*N*	Mean	SD	Median	IQR
UIC, μg/l^a^	72	82	39	76	55–100
Estimated iodine intake, I-screener, μg/d^b^*	72	128	67	124	84–161
Estimated iodine intake, 24HR, μg/d^c^*	72	202	199	136	87–214
Estimated iodine intake, E-UIC^c^, (μg/d)^d^*	72	127	66	112	79–170

a Pooled sample of spot-urine samples from six consecutive days, one participant was missing one spot-urine sample (day 6).

b Iodine-specific digital dietary screener (I-screener).

c Iodine intake from 24-hour recall (24HR).

d Iodine intake estimated with the equation: Urinary iodine concentration (μg/l) × 0⋅0235 × body weight (kg) (10) Self-reported current body weight used for estimation.

* Differences between the different methods were tested by Wilcoxon's signed-rank test. Difference between estimated iodine intake from I-FFQ and 24-hour recall: *P* = 0⋅007; estimated iodine intake from I-FFQ and UIC: *P* = 0⋅969; estimated iodine intake from 24-hour recall and UIC: *P* = 0⋅014.

### Estimated daily iodine intake

The estimated iodine intake from the I-screener, 24HR and estimated iodine intake from UIC in the study population is shown in Table 3. The median estimated iodine intake from the I-screener was 124 μg/d, which was significantly lower than the 24HR (*P* = 0⋅007). The median estimated iodine intake from the 24HR was 136 μg/d and was the method assessing that estimated the highest iodine intake. The median estimated iodine intake based on UIC was 112 μg/d and was significantly lower than the 24HR (*P* = 0⋅014), while no significant difference was found between the estimated iodine intake from UIC and estimated iodine intake from the I-screener (*P* = 0⋅969). The I-screener placed fifty-three participants (74 %) below the dietary iodine recommendations (150 μg/d), whereas the 24HR and estimated iodine intake from UIC placed 39 (54 %) and 51 (71 %) below the recommendations, respectively (Supplementary Table S2).

### Comparison of the I-screener, 24HR and UIC

The correlation between iodine intake from the I-screener, 24HR and estimated intake from UIC, and UIC is presented in Table 4. The only significant correlation was found between the I-screener and the 24HR (*P* = 0⋅006) with a correlation coefficient of 0⋅318, which is considered an acceptable level of correlation^(24)^. A sensitivity analysis was performed removing one extreme value of 1200 μg iodine/d from the analysis. The correlation coefficient remained in the same magnitude of 0⋅292 and the correlation remained significant (*P* = 0⋅014) (see Supplementary Table S3).

**Table 4.** Spearman's rho correlation coefficient between estimated iodine intake from I-screener, 24-hour recall, estimated iodine intake from UIC (E-UIC) and urinary iodine concentration (UIC) (μg/l)

Estimated iodine intake from	24HR^a^ (*n* 72)	E-UIC^b^ (*n* 72)	UIC^c^ (*n* 72)
I-screener^d^	0⋅318*	0⋅140	0⋅122
24HR	–	0⋅209	0⋅165
E-UIC	0⋅209	–	–

Spearman's rank order correlation coefficient. The correlation coefficients strength (effect size) was considered poor if <0⋅20, acceptable if 0⋅20–0⋅49, and strong if ≥0⋅50 corresponding to previously used dietary methods (45).

a Iodine intake from 24-hour recall (24HR).

b Iodine intake estimated with the equation (E-UIC): Urinary iodine concentration (μg/l) × 0⋅0235 × body weight (kg) (21) Self-reported current body weight used for estimation.

c Urinary iodine concentration (UIC): Pooled sample of spot-urine samples from six consecutive days, one participant was missing one spot-urine sample (day 6).

d Iodine-specific digital dietary screener (I-screener).

* Significant correlation coefficient.

The stability of tertile membership between the different methods is presented in Table 5. The stability was highest between the estimated iodine intake from I-screener and the 24HR, where 46 % were classified into the same tertile, 83 % in the stable/adjacent tertile and 17 % in the opposite tertile showing a slight agreement (*k* = 0⋅187). A slight agreement was also seen between the I-screener and UIC (*k* = 0⋅187) and between the I-screener and estimated iodine intake from UIC (*k* = 0⋅229).

**Table 5.** Agreement of tertile membership between estimated iodine intake from the iodine-specific digital dietary screener (I-screener) and the 24-hour recall, with estimated iodine intake from UIC (E-UIC), and urinary iodine concentration (UIC).

	I-screener *v.* 24HR (*n* 72)	I-screener *v.* E-UIC (*n* 72)	I-screener *v.* UIC (*n* 72)	24HR *v.* E-UIC (*n* 72)	24HR *v.* UIC (*n* 72)
Stable tertile (%)	46	49	46	43	36
Stable/adjacent (%)	83	78	78	81	36
Opposite tertile (%)	17	19	22	18	19
Cohen's kappa	0⋅187	0⋅229	0⋅187	0⋅146	0⋅042

**Fig. 3.**
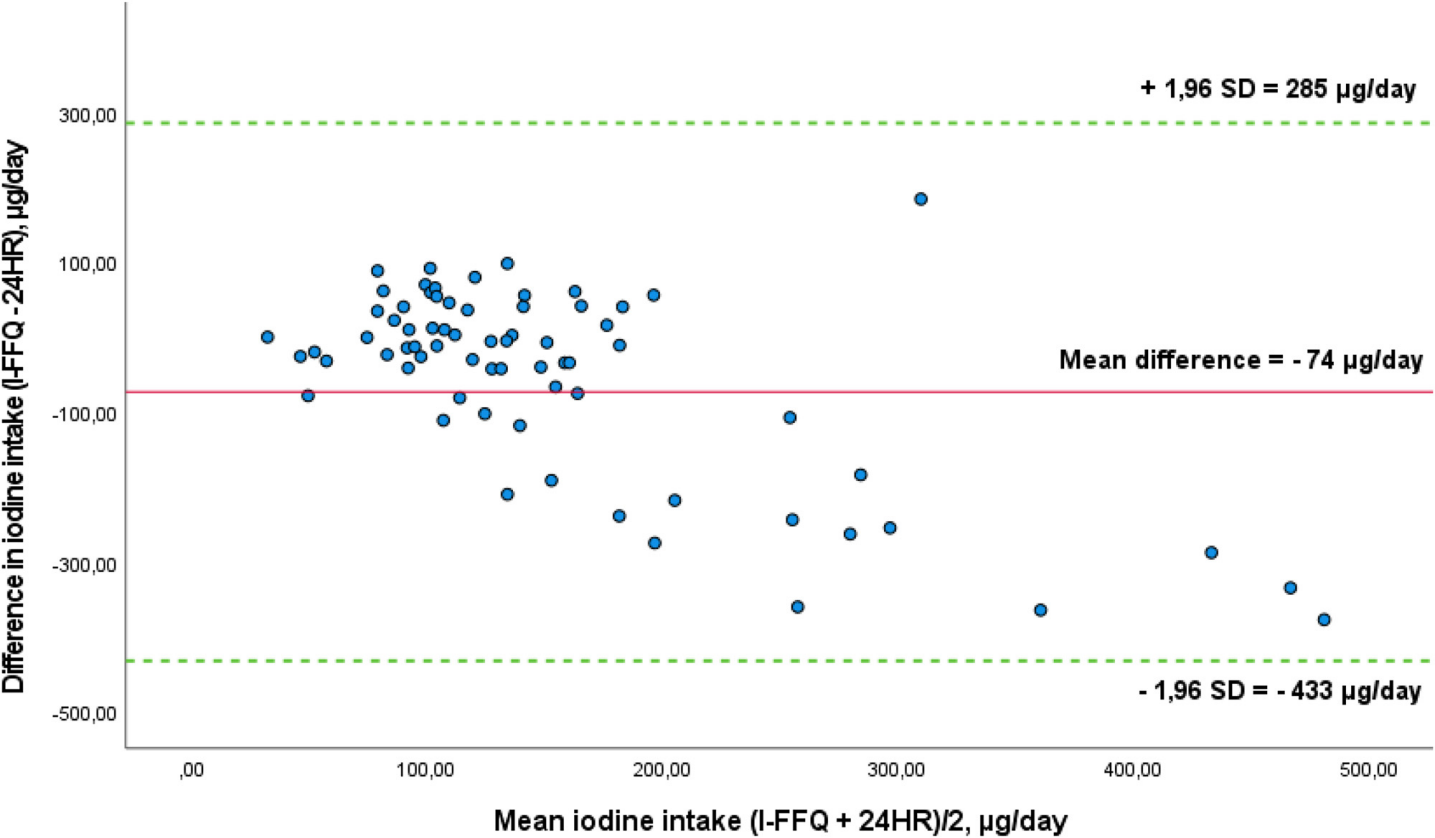
Bland–Altman plot of agreement between the estimated iodine intake from the I-screener and estimated iodine intake from 24-hour recall (24HR) (*n* 72). The solid red line represents the mean difference between the two methods (−74 μg/d), and the dotted green lines represent the limits of agreement (LOA) corresponding to ±1⋅96 standard deviations (sd) (lower agreement: −433 μg/d, upper agreement: 285 μg/d). Two outliers are outside of the plot.

The Bland–Altman plot comparing estimated iodine intake from the I-screener, and the 24HR is presented in Fig. 3. The mean absolute difference in iodine intake between the methods was observed to be −74 μg/d. The LOA ranged from −433 (lower) to 285 (upper) μg/d. Two out of seventy-two (3 %) participants were found to lie beyond the LOA. The difference in estimated iodine intake between the methods seems to increase with higher intake, seen as the scatter widens with increasing mean values. The estimated iodine intake was higher from the 24HR compared to the I-screener.

**Fig. 4.**
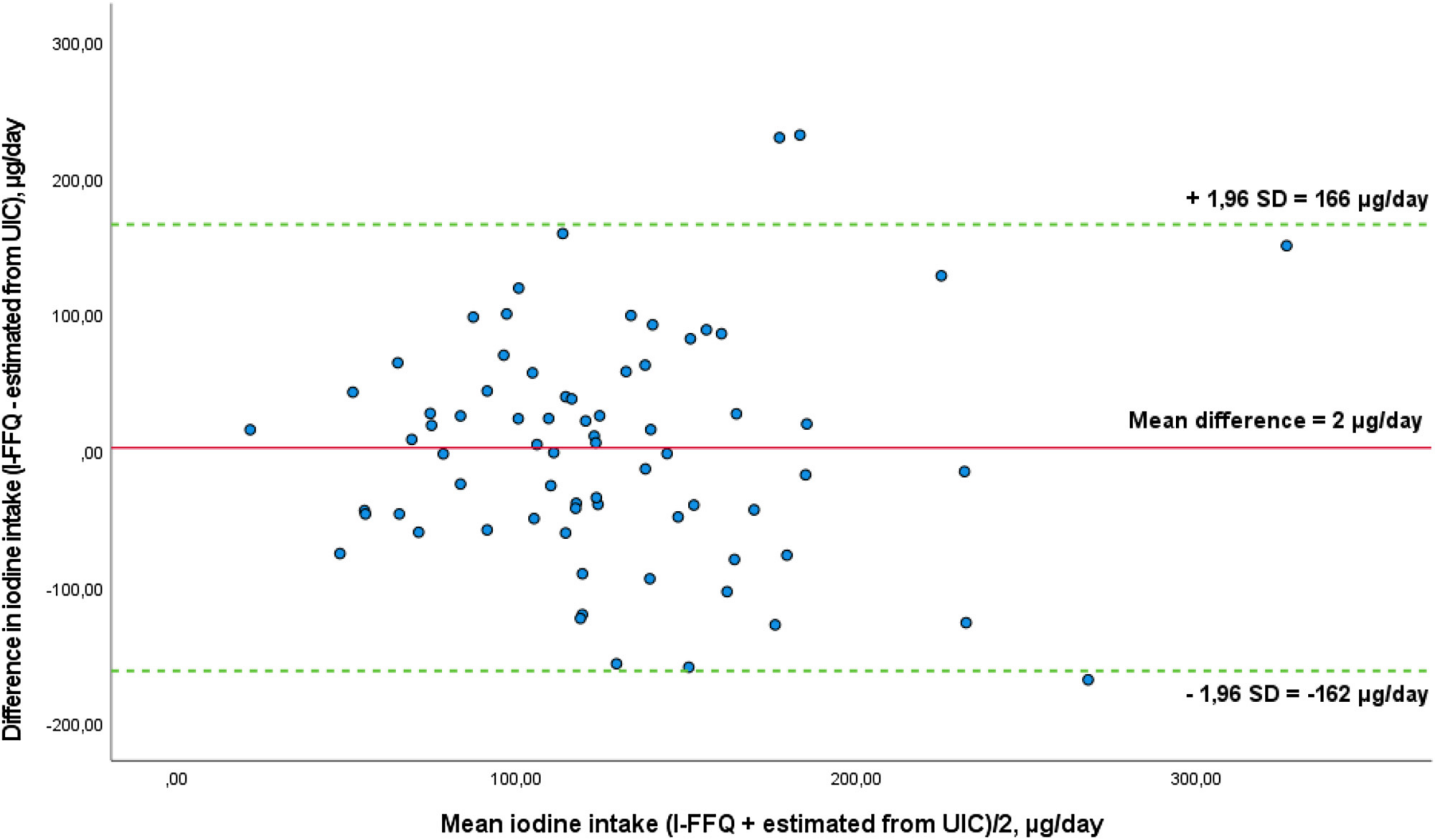
Bland–Altman plot of agreement between estimated iodine intake from the iodine-specific digital dietary screener (I-screener) and the estimated iodine intake from UIC (*n* 72). The solid red line represents the mean difference between the two methods (2 μg/d), and the dotted green lines represent the limits of agreement (LOA) corresponding to ± 1⋅96 standard deviations (SD) (lower agreement: −162 μg/d, upper agreement: 166 μg/d).

The Bland–Altman plot comparing estimated iodine intake from the I-screener and UIC is presented in Fig. 4. The mean absolute difference in iodine intake between the methods was observed to be 2 μg/d. The LOA ranged from −162 (lower) to 166 (upper) μg/d. Three out of seventy-two (4 %) participants were found to lie beyond the LOA. Based on these results, one can assume an agreement between the estimated iodine intake from the I-screener and the estimated intake from UIC as the results are in the same magnitude irrespective of intake quantity.

**Fig. 5.**
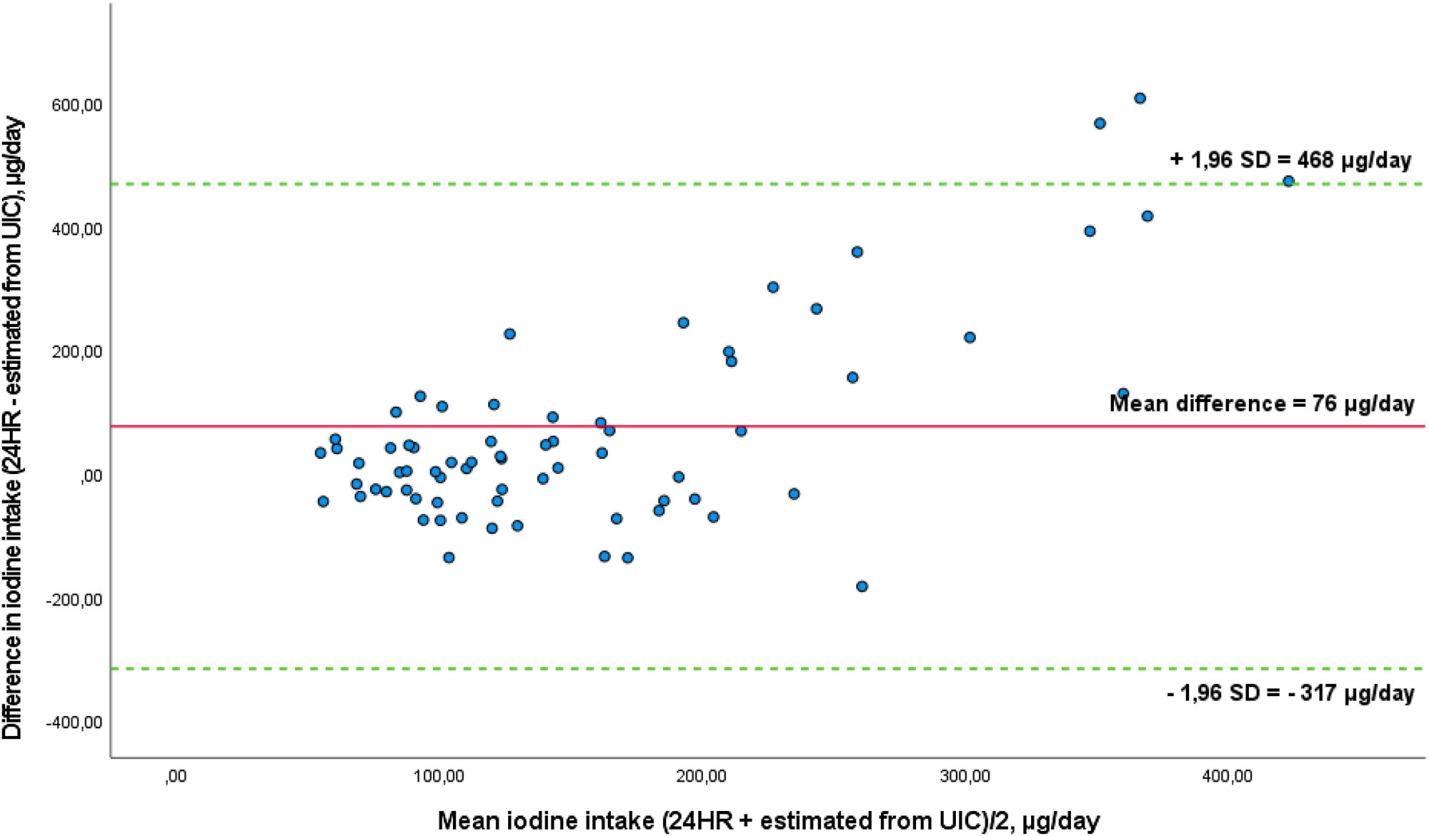
Bland–Altman plot of agreement between estimated iodine intake from the 24-hour recall (24HR) and estimated iodine intake from UIC (*n* 72). The solid red line represents the mean difference between the two methods (−76 μg/d), and the dotted green lines represent the limits of agreement (LOA) corresponding to ±1⋅96 standard deviations (SD) (lower agreement: −317 μg/d, upper agreement: 468 μg/d). One outlier is outside the figure.

The Bland–Altman plot comparing estimated iodine intake from the 24HR and UIC is presented in Fig. 5. The mean absolute difference in iodine intake between the methods was observed to be 76 μg/d. The LOA ranged from −317 (lower) to 468 (upper) μg/d. Four out of seventy-two (6 %) participants were found to lie beyond the LOA. The scatter widens with increasing mean values, showing that the difference in estimated iodine intake between the methods seems to increase with higher intake. The estimated iodine intake was higher from the 24HR compared to the estimated iodine intake from UIC. Removing the extreme value of 1200 μg iodine/d did not change the agreement between the methods (data not shown).

## Discussion

The present comparison study in women of reproductive age comparing iodine intake estimates from the I-screener against 24HR and UIC from six spot-urine samples found an acceptable correlation between I-screener and 24HR, but not between I-screener and UIC. There have previously been performed comparison studies for iodine-specific FFQs (I-FFQs) specifically targeting Norwegian pregnant women^(25,26)^, however, no shortform of FFQ or I-screeners have been validated for the Norwegian female population of reproductive age.

Previous investigation of young women in Norway suggests an overall mild iodine deficiency in this population group^(3,27)^. This is in line with the results in the present study population, which found that the medians of the three iodine intake estimates are all below the recommended iodine intake of 150 μg/d.

In previous comparison studies of I-FFQs, some have observed higher estimates of iodine intake from the dietary reference method^(13,28)^, while others have found the I-FFQ to estimate a higher iodine intake compared to the reference method^(26,29)^. There could be several reasons why the 24HR in the present study estimated a higher median iodine intake than the I-screener. The 24HR was only conducted once and did therefore not capture the day-to-day variation of iodine-rich dietary sources. As the calculation from the 24HR recall included all foods consumed, the 24HR could have estimated a higher iodine intake due to the possible contribution of other food groups to the iodine intake estimate not included in the I-screener. The I-screener only consists of thirteen iodine items focusing on the main sources of dietary iodine intake. Furthermore, a single 24HR may be low in precision, especially among the ‘high iodine consumers’ for whom white marine fish was the main contributor to iodine intake. The food composition data for white marine fish is also known to be variable in quality^(30)^, which could lead to imprecision in the iodine intake estimates.

The strongest correlation was found between the I-screener and 24HR, which is considered acceptable^(24)^. This correlation was lower than what has been found in similar studies using food diaries or 3–4 d food records^(13,26,28,31)^, but higher than in a validation study using a 3-d dietary record^(29)^. As the present study used 24HR as a dietary reference, a lower correlation was expected due to the difference in the period of assessment between the methods. The correlation between the I-screener and UIC was poor but increased slightly when estimated iodine intake from UIC was assessed (*r* = 0⋅140). Næss *et al.*^(26)^ who used the same method for urine sampling found an acceptable significant correlation between an I-FFQ and UIC (*r* = 0⋅21). Nonetheless, correlation coefficients only reflect the strength and direction of the association and do not measure agreement between the methods. Hence, it is not appropriate to use it as a separate determinant of validity^(32)^.

The agreement between the I-FFQ and 24HR recall in the present study was fair. The agreement between methods was assessed in two other I-FFQ studies^(26,28)^. One of the studies found a moderate to substantial agreement between their I-FFQ and the dietary reference method^(26)^. While the other found a fair agreement between the dietary reference methods comparable to the present study^(28)^. As an acceptable correlation was observed between the I-screener and 24HR, it strengthens the findings of an agreement between the dietary assessment methods in the present study^(32)^.

The agreement between the I-screener and UIC in the present study was found to be weak, with 46 % allocated in the same tertile (*k* = 0⋅147), which is slightly lower than in comparable studies^(26,28)^, but similar to the findings in another study^(33)^. In our study, we also assessed the agreement between the I-screener and estimated iodine intake from UIC, which showed a slightly stronger agreement. A biomarker can provide an objective measure of iodine status, although UIC is not considered a gold standard biomarker for individual iodine status due to the large day-to-day variation in iodine intake. However, a strength of the present study was that UIC was analysed in a pooled sample of spot urine from six consecutive days, which may reduce the day-to-day variability of the measure. As the I-screener estimates the iodine intake based on a habitual weekly intake, six spot-urine samples cover a similar period which is preferred when conducting comparison studies^(14)^. However, the agreement between I-screener and UIC from six spot-urine samples was found to be poor. This could be explained by the accuracy of UIC as a biomarker, as at least ten spot-urine samples or 24-h urine collections are suggested to account for inter- and intraindividual variability^(34,35)^. Accounting for the burden of these methods for the participants, this would not be feasible in most studies including the present one. However, we cannot rule out that the estimated intake from the I-screener is the cause of the lack of agreement between these two methods.

A strength of the present study is the use of two reference methods, both 24HR and UIC. The comparison between the methods was also assessed with multiple statistical methods, as recommended. The sample size was considered sufficient for a dietary screener comparison study^(14)^. The completion rate was remarkably high (91 %), which indicates that the study was feasible for the participants. A recent study on the present I-screener assessed the completion rate ‘in a real-life setting’. The completion rate was high, showing that the length does not extend beyond the participant's willingness and is a feasible tool^(19)^.

A few limitations of the study should be outlined. First, the generalisability of the target population should be mentioned. Based on their UIC, BMI and tobacco use, the population was comparable to the general Norwegian females^(3,27,36,37)^. The education level was, however, higher compared to the general Norwegian female population^(38)^. The study population was recruited from one geographical area in Norway (Bergen). However, the main threat to the external validity of this study is the high percentage of subjects in health-related occupations. Considering that the study population is homogeneous, and the known lack of association between iodine status, socioeconomic status and geographical areas^(5,39,40)^, it is believed that the present results may apply to the target population. Second, the dietary reference method was a single 24HR and not repeated assessment of intake. In addition, compared to other comparability studies of I-FFQs, the present I-screener is a short dietary screener consisting of only thirteen items^(13,26,29,31,33)^. This could lead to an underestimation of iodine intake, however, as the sources of iodine in the Norwegian diet are few, the number of included items seems to capture the main sources though at a highly aggregated level. Weighted dietary records or diet records are considered the preferable reference method for the FFQ as they can reflect the same dietary intake period as the FFQ^(14)^. The use of a single 24HR as the reference dietary method was chosen to make participation in the study feasible. Third, the present I-screener stratifies the user into three categories of iodine intake in contrast to others that estimate an iodine intake value^(13,26,28)^. This makes a comparison to similar studies challenging. Fourth, self-reported body weight was used in the E-UIC calculation, and might influence the accuracy of this estimate as would the lack of adjustment for u-creatine that was not available. Last, an assessment of reproducibility should always be applied in comparison studies^(14)^, however, this was not possible in the present study due to limited time and resources.

## Conclusion

The present study shows an acceptable correlation and agreement between the I-screener and 24HR, but not with the UIC from six spot-urine samples. As a cost- and time-efficient tool, the I-screener is a promising tool for indicating inadequate iodine intake. As misclassification in iodine intake between the different methods were relatively prevalent, the screening tool is not to be used for diagnostic purposes, and should therefore be used with caution. Further research is needed for assessing reproducibility and validity of the I-screener in different population groups.

## Supplementary material

For supplementary material accompanying this paper visit https://doi.org/10.1017/jns.2023.74. click here to view supplementary material
