# Supplementary material for: Comparison of a digital iodine-specific dietary screener with 24-hour recall and urinary iodine concentration
Source: J Nutr Sci. 2023 Aug 1;12:e90. doi: 10.1017/jns.2023.74 (PMC10427488; doi:10.1017/jns.2023.74)
Supplement: Supplementary file 1 [file S2048679023000745sup001.docx]

**Supplementary Table 1:** An overview of the dietary items and questions in the iodine-specific digital dietary screener (I-screener).

| **English translation** | **Norwegian (original language)** |
| --- | --- |
| Who should you test iodine status for?  - Myself  - My child | Hvem skal du teste jodstatus for?   - Meg selv - Barnet mitt |
| Gender and age  - Female 18 years or older  - Male 18 years or older  - Girl/boy 10-17 years | Kjønn og alder   - Kvinne 18 år eller eldre - Mann 18 år eller eldre - Jente/gutt 10-17 år |
| About you.  - I plan to get pregnant  - I'm pregnant  - I'm breastfeeding  - Neither | Om deg.   - Jeg planlegger å bli gravid - Jeg er gravid - Jeg ammer - Ingen av delene |
| How much milk, chocolate milk, and sour milk do you drink?  - 3 glasses or more per day  - 1-2 glasses per day  - 4-6 glasses a week  - 1-3 glasses a week  - Rarely or never drinks milk | Hvor mye melk, sjokolademelk og syrnet melk drikker du?   - 3 glass eller mer per dag - 1-2 glass per dag - 4-6 glass i uken - 1-3 glass i uken - Drikker sjelden eller aldri melk |
| How many cups of coffee/tea with a lot of milk (1.7 dl) do you drink (eg caffè latte, cappuccino, chai latte)?  - 2 cups or more per day  - 1 cup per day  - 4-6 cups a week  - 1-3 cups a week  - Rarely or never drinks coffee/tea with milk | Hvor mange kopper kaffe/te med mye melk (1,7 dl) drikker du (eks. caffè latte, cappuccino, chai latte)?   - 2 kopper eller flere per dag - 1 kopp per dag - 4-6 kopper i uken - 1-3 kopper i uken - Drikker sjelden eller aldri kaffe/te med melk |
| How many portions of cereal and/or porridge made with milk (1.5 dl) do you eat?  - 2 or more portions per day  - 1 portion per day  - 4-6 portions a week  - 1-3 portions a week  - Rarely or never eats cereal/porridge with milk | Hvor mange porsjoner frokostblanding og/eller grøt laget med melk (1,5 dl) spiser du?   - 2 eller flere porsjoner per dag - 1 porsjon per dag - 4-6 porsjoner i uken - 1-3 porsjoner i uken - Spiser sjelden eller aldri frokostblanding/grøt med melk |
| How many slices of bread/crispy bread with prim do you eat?  - 2 or more per day  - 1 per day  - 4-6 a week  - 1-3 a week  - Rarely or never prime | Hvor mange brødskiver/knekkebrød med prim spiser du?   - 2 eller flere per dag - 1 per dag - 4-6 i uken - 1-3 uken - Sjelden eller aldri prim |
| How many slices of bread/crispy bread with brown cheese do you eat?  - 2 or more per day  - 1 per day  - 4-6 a week  - 1-3 a week  - Rarely or never brown cheese | Hvor mange brødskiver/knekkebrød med brunost spiser du?   - 2 eller flere per dag - 1 per dag - 4-6 i uken - 1-3 uken - Sjelden eller aldri brunost |
| How many portions of yogurt or quark do you eat (125 g, incl. Skyr and Kesam)?  - 2 or more portions per day  - 1 portion every day  - 4-6 portions a week  - 1-3 portions a week  - Rarely or never eats yoghurt or quark | Hvor mange porsjoner yoghurt eller kvarg spiser du (125 g, inkl. Skyr og Kesam)?   - 2 eller flere porsjoner per dag - 1 porsjon hver dag - 4-6 porsjoner i uken - 1-3 porsjoner i uken - Spiser sjelden eller aldri yoghurt eller kvarg |
| How many portions of cottage cheese do you eat (100 g)?  - 2 or more portions per day  - 1 portion every day  - 4-6 portions a week  - 1-3 portions a week  - Rarely or never eats cottage cheese | Hvor mange porsjoner cottage cheese spiser du (100 g)?   - 2 eller flere porsjoner per dag - 1 porsjon hver dag - 4-6 porsjoner i uken - 1-3 porsjoner i uken - Spiser sjelden eller aldri cottage cheese |
| How often do you eat pure white fish (200 g, not mixed products)?  - 2-3 times a week or more  - 1 time a week  - 2-3 times a month  - 1 time a month  - Rarely or never eats white fish | Hvor ofte spiser du ren hvit fisk (200 g, ikke blandingsprodukter)?   - 2-3 ganger i uken eller mer - 1 gang i uken - 2-3 ganger i måneden - 1 gang i måneden - Spiser sjelden eller aldri hvit fisk |
| How often do you eat white fish products (150 g, fish cakes, fish pudding, fish sticks, etc.)?  - 2-3 times a week or more  - 1 time a week  - 2-3 times a month  - 1 time a month  - Rarely or never eat white fish products | Hvor ofte spiser du produkter av hvit fisk (150 g, fiskekaker, fiskepudding, fiskepinner osv.)?   - 2-3 ganger i uken eller mer - 1 gang i uken - 2-3 ganger i måneden - 1 gang i måneden - Spiser sjelden eller aldri produkter av hvit fisk |
| How many slices of bread/crispy bread with fish toppings such as mackerel in tomato and caviar do you eat?  - 2 or more times per day  - 1 per day  - 4-6 a week  - 1-3 a week  - Rarely or never eats fish dishes | Hvor mange brødskiver/knekkebrød med fiskepålegg som makrell i tomat og kaviar spiser du?   - 2 eller flere ganger pr dag - 1 per dag - 4-6 i uken - 1-3 i uken - Spiser sjelden eller aldri fiskepålegg |
| Hvor mange egg spiser du (inkl. i matlaging)?   - 1 eller flere egg per dag - 4-6 egg i uken - 1-3 egg i uken   Spiser sjelden eller aldri egg | Hvor mange egg spiser du (inkl. i matlaging)?   - 1 eller flere egg per dag - 4-6 egg i uken - 1-3 egg i uken - Spiser sjelden eller aldri egg |
| How often do you use oat milk that has added iodine (1.5 dl)?  - 3 times a day  - 1-2 times a day  - 4-6 times a week  - 1-3 times a week  - Rarely or never uses plant-based milk | Hvor ofte bruker du havredrikk som er tilsatt jod (1,5 dl)?   - 3 ganger om dagen - 1-2 ganger om dagen - 4-6 ganger i uken - 1-3 ganger i uken - Bruker sjelden eller aldri plantedrikker |
| Do you use dietary supplements containing iodine (150 µg)?  - Yes, daily  - About every other day  - Occasionally (once a week)  - Rarely or never | Bruker du kosttilskudd som inneholder jod (150 µg) ?   - Ja, daglig - Ca. annenhver dag - Av og til (en gang i uken) - Sjelden eller aldri |

**Supplementary Table 2:** Overview of the participants with estimated iodine intake below the dietary iodine recommendations of 150 µg/day.

| **Estimated iodine intake from, n (%)** | **Number of participants with iodine intake below 150 µg/day** |
| --- | --- |
| **I-screener^a^** | 53 (74%) |
| **24HR^b^** | 39 (54%) |
| **E-UIC^c^** | 51 (71%) |

^a^ Iodine-specific digital dietary screener (I-screener)

^b^ Iodine intake from 24-hour recall (24HR)

^c^ Iodine intake estimated with the equation (E-UIC): Urinary iodine concentration (μg/L) × 0.0235 × body weight (kg) [21] Self-reported current body weight used for estimation.

**Supplementary Table 3:** Spearman´s rho correlation coefficient^a^ between estimated iodine intake from I-screener, 24-hour recall, estimated iodine intake from UIC (E-UIC), urinary iodine concentration (UIC) (ug/L) and Tg (ug/L) without an extreme outlier of 1200 µg iodine/d.

| Estimated iodine intake from | 24HR (n=72)^c^ | E-UIC (n=72)^d^ | UIC (n=72)^e^ | Tg (n=61) |
| --- | --- | --- | --- | --- |
| I-screener^b^ | 0.292* | 0.124 | 0.116 | 0.189 |
| 24HR | - | 0.187 | 0.157 | -0.075 |
| E-UIC | 0.209 | - | - | 0.053 |

Tg, thyroglobulin

^a^ Spearman´s rank order correlation coefficient. The correlation coefficients strength (effect size) was considered poor if < 0,20, acceptable if 0,20-0,49, and strong if > 0,50 corresponding to previously used dietary methods [45]

^b^ Iodine-specific digital dietary screener (I-screener)

^c^ Iodine intake from 24-hour recall (24HR)

^d^ Iodine intake estimated with the equation (E-UIC): Urinary iodine concentration (μg/L) × 0.0235 × body weight (kg) [21] Self-reported current body weight used for estimation.

^e^ Urinary iodine concentration (UIC): Pooled sample of spot-urine samples from six consecutive days, one participant was missing one spot-urine sample (day 6)

^*^ Significant correlation coefficient
